# Myoblast fusion confusion: the resolution begins

**DOI:** 10.1186/s13395-017-0149-3

**Published:** 2018-01-31

**Authors:** Srihari C. Sampath, Srinath C. Sampath, Douglas P. Millay

**Affiliations:** 1 Genomics Institute of the Novartis Research Foundation, 10675 John Jay Hopkins Drive, San Diego, CA 92121 USA; 2 Division of Musculoskeletal Imaging, Department of Radiology, University of California San Diego School of Medicine, 200 West Arbor Drive, San Diego, CA 92103 USA; 3 Department of Molecular Cardiovascular Biology, Cincinnati Children’s Hospital Medical Center, 240 Albert Sabin Way, Cincinnati, OH 45229 USA; 4 Department of Pediatrics, University of Cincinnati College of Medicine, Cincinnati, OH 45229 USA

**Keywords:** Myoblast fusion, Myomaker, Myomerger, Minion, Muscle development

## Abstract

The fusion of muscle precursor cells is a required event for proper skeletal muscle development and regeneration. Numerous proteins have been implicated to function in myoblast fusion; however, the majority are expressed in diverse tissues and regulate numerous cellular processes. How myoblast fusion is triggered and coordinated in a muscle-specific manner has remained a mystery for decades. Through the discovery of two muscle-specific fusion proteins, Myomaker and Myomerger–Minion, we are now primed to make significant advances in our knowledge of myoblast fusion. This article reviews the latest findings regarding the biology of Myomaker and Minion–Myomerger, places these findings in the context of known pathways in mammalian myoblast fusion, and highlights areas that require further investigation. As our understanding of myoblast fusion matures so does our potential ability to manipulate cell fusion for therapeutic purposes.

## Background

Muscle formation occurs during several stages of an organism’s life, including embryonic development, and growth and regeneration in the adult. Proper myogenesis begins through specification of precursor cells to the myoblast lineage, followed by differentiation, both of which are accomplished by the muscle-specific transcription factors MyoD and Myogenin [1, 2]. A critical event in myogenesis is the fusion of myoblasts either with one another to generate new multi-nucleated myofibers or with an existing myofiber, thereby increasing the pool of myonuclei and allowing muscle growth. There has been a relative lack of understanding about the mechanism and regulation of myoblast fusion, when compared to other events that precede it, such as lineage specification and differentiation. Furthermore, it has historically been difficult to uncouple defects in differentiation from those affecting fusion. This is principally because until recently, proteins directly activating fusion but not differentiation remained unidentified, and therefore, methods to dominantly reconstitute fusion and dissect its mechanisms were nonexistent.

Plasma membrane fusion is a complex process that requires recognition, adhesion, cell signaling, cytoskeletal alterations, and membrane rearrangements. Current evidence, reviewed elsewhere, implicates numerous proteins and regulatory pathways in the recognition, adhesion, and the cell signaling phases of myoblast fusion [3]. However, none of these proteins have been shown to serve as a nodal regulator of myoblast membrane fusion. Such a regulator would be expected to be expressed specifically in the muscle, be genetically required for muscle formation, and function to dominantly induce fusion. In addition, fusogens from other systems are characteristically able to render normally non-fusing cells fusogenic. This is the case, for example, of the H and F proteins of paramyxoviruses such as measles virus [4] and the cellular fusogen epithelial fusion failure (Eff-1) in *Caenorhabditis elegans* [5].

Until very recently, skeletal muscle fusogens fulfilling these criteria had not been identified in any species. It was in fact a major conundrum whether muscle-specific proteins that directly governed myoblast fusion actually existed. The discovery of the multi-pass transmembrane protein Myomaker as the first muscle-specific fusion factor was the first step toward resolving the confusion surrounding the question of vertebrate myoblast fusion [6]. Notably, Myomaker could induce fusion of fibroblasts with muscle cells, but not between fibroblasts themselves, highlighting the likelihood that additional myogenic fusion factors existed. Indeed, three independent groups recently identified a second muscle-specific fusion protein named Myomerger–Minion–Myomixer, which when co-expressed with Myomaker is sufficient to induce fusion in non-fusogenic fibroblasts [7–9]. There is now little doubt as to the existence of muscle-specific fusion proteins. However, confusion remains about the mechanisms by which Myomaker and Minion–Myomerger coordinate and accomplish membrane coalescence. The overall goals of this review are to highlight the recent advances in the identification of the myoblast fusion machinery, discuss areas that are currently unresolved, and suggest areas for future research as well as translational application.

## Expression and conservation of Myomaker and Minion–Myomerger in myogenesis

Both Myomaker and Myomerger–Minion were identified through bioinformatic in silico searches. It is surprising that prior to their discovery, the numerous genetic screens performed in *Drosophila*, zebrafish, and C2C12 myoblasts did not yield muscle-specific proteins directly governing myoblast fusion. One possibility for the elusiveness of these genes could be their inadequate annotation, particularly as Minion–Myomerger represents a small ORF (smORF) encoded microprotein which is below the 100 amino acid thresholds normally used to bioinformatically predict open reading frames [10]. Another complicating factor is that both genes are dynamically expressed, with no detectable expression in proliferating myoblast cultures but strong transcriptional induction upon differentiation. As expected based on this pattern, numerous E-box elements have been found upstream of both Myomaker and Myomerger–Minion indicating a role for the myogenic regulatory factors (MRFs), MyoD and Myogenin, in the activation of these genes [11].

In vitro, these genes are expressed in both myocytes (differentiated mono-nucleated myoblasts) and myotubes. This contrasts with what is found in vivo where Myomaker and Myomerger–Minion are expressed in the developing myotome beginning at embryonic (E) day 10 but no longer detectable after postnatal (P) day 21, when muscle development has ceased. In healthy adult myofibers, Myomaker and Minion–Myomerger transcripts or protein have not been detected. However, upon muscle injury, both genes are activated as muscle stem cells (MuSC) differentiate. Additionally, expression of Myomaker and Myomerger–Minion has not been detected in the other mammalian cell types that fuse such as osteoclasts, sperm-egg, trophoblasts, and multinucleated giant cells. This raises the question of whether homologous proteins perform a similar function in other mammalian fusogenic cells and to what extent completely distinct pathways (e.g., syncytin in placental trophoblast) activate fusion in the various cell types.

Myomaker function is conserved to zebrafish where it is expressed only in the fusion competent fast myocytes but not in the fusion-incompetent slow myocytes, and this expression is functional as myomaker deletion results in a lack of fast myocyte fusion [12, 13]. Myomaker function also appears conserved to humans, and mutation of the human Myomaker homolog results in defective myoblast fusion and congenital myopathy [14]. Expression of the zebrafish Myomerger–Minion ortholog in cell-based assays indicates similar function, and indeed, a recent study has shown that it is required for myoblast fusion in vivo [9, 15]. In contrast to the conservation of Myomaker and Minion–Myomerger in zebrafish, whether they are also present in *Drosophila* and other invertebrates is currently unknown. It is unlikely but formally possible that sequence-diverged homologs of Myomaker and Minion–Myomerger remain to be discovered in invertebrates. The more likely scenario is that invertebrates rely on functional homologs that lack sequence similarity. In this case, functional screening of *Drosophila* cDNAs in mammalian cells (for instance using the reconstitution system described below) may allow identification of such proteins. Alternatively, the basic biophysical mechanisms for fusion could be different between the species and thus require different factors to accomplish membrane coalescence.

## Function of Myomaker and Myomerger–Minion in fusion, and reconstitution in non-fusogenic cells

In the mouse, genetic loss-of-function experiments for Myomaker and Minion–Myomerger have revealed that these proteins are necessary for muscle formation. Indeed, loss of either gene results in death at birth due to a lack of skeletal muscle, and defects are seen in muscles of both somitic and non-somitic origin. Of note, mono-nucleated myosin^+^ cells were detected in both strains of mice during development, indicating an ability to differentiate and thus demonstrating that loss of these genes do not directly regulate differentiation. Cultured myoblasts from each genetically modified mouse line also displayed the ability to differentiate, but not fuse, confirming a highly specific role for these proteins in fusion.

Although such loss-of-function studies have identified proteins required for muscle cell fusion in a variety of systems, the ultimate demonstration of fusogenic function would come from gain-of-function studies, particularly reconstitution of fusogenic activity in otherwise non-fusogenic cells. The identification of Myomaker represented a significant step toward this goal, in that fibroblast cells expressing Myomaker were capable of robustly fusing to differentiating C2C12 myoblasts—a surprising and dramatic demonstration that Myomaker expression alone is sufficient to confer fusion competence to non-muscle cells. The observation that Myomaker-expressing fibroblasts never fused with one another led to the hypothesis of another activity required for cell fusion, one that could induce fusogenicity by driving pore formation and expansion. The search for this elusive fusion-inducing activity culminated in the identification of Minion–Myomerger. Data from several groups has now demonstrated that co-expression of Myomaker and Minion–Myomerger is sufficient to drive fusion of otherwise non-fusogenic cells [7–9]. This process is both rapid and efficient; fibroblasts infected with retroviruses encoding these proteins begin to fuse within hours, eventually forming massive syncytial conglomerates. Few if any infected but unfused cells are observed.

From an experimental perspective, the minimal two-component nature of this program offers the chance to investigate mechanisms through gain of function and cell mixing studies. For instance, mixing of differentially labeled populations revealed an intriguing asymmetry: mouse fibroblasts expressing both Myomaker and Minion–Myomerger were capable of fusing to myoblasts expressing Myomaker alone [7, 8]. Stated differently, Myomaker was required symmetrically (on both fusing cells), whereas Minion/Myomerger was required asymmetrically (only one cell of the pair). This asymmetry is also present when Myomaker and Myomerger–Minion are overexpressed in myoblasts in growth medium [7], and is supported by knockout studies showing that whereas Minion–Myomerger-deficient myoblasts efficiently fuse to wild-type myoblasts, Myomaker-deficient myoblasts do not [7, 9]. Extrapolating this phenomenon to multiple rounds of fusion, it becomes clear that expression of Minion–Myomerger in a single cell among a population of otherwise homogeneous Myomaker-expressing cells could in principle lead to the formation of a new myotube. This would of course define a fusion mechanism analogous to that seen in *Drosophila* myogenesis, with cells expressing both Myomaker and Minion–Myomerger playing the role of “founder” myoblasts and those expressing only Myomaker serving as “fusion competent” myoblasts.

Although such a mechanism would reconcile the otherwise disparate-appearing fusion mechanisms of flies and mammals, it must be emphasized that there is currently no in vivo experimental evidence to support the existence of such myoblast heterogeneity in mammals. On the contrary, it has been shown that both the Myomaker and Minion–Myomerger genes contain evolutionarily conserved E-box binding sites for canonical myogenic regulatory factors such as MyoD and Myogenin; these basic helix-loop-helix transcription factors orchestrate expression of a large number of genes required for myogenesis, suggesting that Myomaker and Minion–Myomerger are indeed coordinately expressed. Minion–Myomerger was also reported as a hit in a dropout screen where presumably only one cell in the fusion pair was lacking the protein [9], suggesting that any asymmetry may not be as strong as it appears in an overexpression context. Finally, Myomaker is necessary in both fusing cells during in vitro (de novo) myogenesis, but only on muscle stem cells and not myofibers during adult muscle growth [16], demonstrating that the presence of asymmetry may depend on the physiological and developmental context. Some of these observations could be reconciled if asymmetry in muscle cell fusion were defined not by expression of Myomaker and Minion–Myomerger per se but by their regulation, either via interacting proteins or post-translational modification. Whether this is indeed the case is an important area for future research.

While the induction of fusion in fibroblasts by expression of Myomaker and Minion–Myomerger demonstrates that these two proteins are sufficient for cellular fusion, additional factors may be involved. One such example is the reported physical association of Minion with cytoskeleton-associated proteins, and the requirement for cytoskeletal remodeling downstream of Myomaker–Minion–Myomerger induced cell fusion [7]. Recent data also demonstrates that Myomaker functions at the plasma membrane but is localized to multiple cellular compartments, suggesting that Myomaker function requires proper trafficking to sites of fusion [17]. These data suggest that Myomaker and Minion–Myomerger together initiate and engage multiple cell biological effector pathways which are more widely expressed in mammalian cells. These mechanisms should now be readily identifiable using CRISPR-based approaches in the heterologous fibroblast model, exemplifying the potential utility of this simplified system. Given the difficulty of distinguishing effects on differentiation from effects on myocyte fusion, modulation of induced fusion should also prove useful as a method for validating new putative fusion regulators.

## Structure and mutagenesis of Myomaker and Minion–Myomerger

Our understanding of the structural characteristics of these myogenic fusion proteins is in its infancy. Given that the structural analyses of the specific fusogenic proteins that mediate viral and intracellular membrane fusion have provided powerful insights into those phenomena, we anticipate that understanding the structure of Myomaker (221 amino acids) and Myomerger–Minion (84 amino acids), and their respective functional domains, will illuminate the mechanisms underlying myoblast fusion.

Overall, Myomaker exhibits minimal homology to other known proteins. Although its primary sequence has modest similarity to Tmem8a and Tmem8b, neither of these orphan membrane proteins possesses the fusogenic activity for fibroblast-myoblast fusion [18]. Therefore, it will likely require extensive experimental strategies to reveal Myomaker’s structure and ultimately its function(s). Immunostaining of live cells expressing FLAG-tagged versions of Myomaker had previously indicated that this protein exhibits seven transmembrane domains with an extracellular N-terminus and intracellular C-terminus [18]. One note of caution with this analysis was that engineering a FLAG tag on Myomaker in any position reduced its fusogenic activity, which raised the possibility of associated perturbations in domain orientation. However, results from more recent live immunostaining assays of wild-type C2C12 cells using a commercially available antibody against wild-type Myomaker were consistent with the model generated from the FLAG-tagged versions [17].

Although the current topology model for Myomaker is not yet complete, it remains informative with respect to potential functions. The majority of the protein is embedded in the membrane with only small regions available in the extracellular space, suggesting that it does not act as a traditional adhesion molecule akin to the Ig superfamily that functions in *Drosophila* myoblast fusion. This does not preclude the potential for Myomaker to act across short distances after adhesion has been initiated by other traditional factors, to bring membranes even closer and promote coalescence. Additionally, regions of the protein not embedded in the membrane extend modestly into the extracellular and intracellular space, suggesting that it could drive fusion by acting in *cis* or *trans*. A requirement for the intracellular C-terminal domain for fusogenic activity of Myomaker suggests that a function in *cis* is required. Deletion of the final seven amino acids (amino acids 215–221) or mutation of these amino acids to alanine results in a complete lack of fusion as assessed by the ability of the mutant protein to fuse fibroblasts to myoblasts or rescue the fusogenic defect in Myomaker knockout myoblasts [18]. The C-terminal region of Myomaker harbors three conserved cysteines that are palmitoylated [17]; however, the exact role for lipidation is not known. It is possible that palmitoylation of Myomaker governs intracellular/Golgi trafficking or placing the protein within the proper membrane microdomain for driving fusion. A similar regulatory mechanism was recently shown to be operational in *C. elegans* to control the fusogen Eff-1 [19].

In contrast to Myomaker, much less is known regarding the structural characteristics of Myomerger–Minion. In silico analysis of secondary structure reveals multiple helical regions that could mediate membrane binding. Portions of this helical region have predicted amphipathic nature (Schmedt and Sampath, unpublished observation) which may influence function, as discussed below. Of these three helical structures, only the N-terminal hydrophobic region (amino acids 5–25) has the required length (18–20 amino acids) to span the membrane bilayer and potentially act as a membrane anchor. Disrupting the positively charged arginines within the first α-helix following the N-terminal hydrophobic domain resulted in a loss of fusogenic activity and perturbed interaction with FLAG–Myomaker as evidenced by co-immunoprecipitation [9].

Of note, the N-terminal hydrophobic region has also been interpreted as a cleavable signal peptide; if true, this would suggest that Myomerger–Minion is secreted and may activate fusion through extracellular interactions. However, subcellular fractionation indicates that the vast majority of the protein sorts with membrane fractions, suggesting that it is tethered to a membrane. A potential longer isoform containing sequence upstream of the signal peptide is also predicted in the mouse. While this species is unlikely to be translated as endogenous Minion matches the molecular weight of the “short” 84 amino acid isoform (Zhang and Sampath, unpublished observation), it is sufficient to fuse myomaker^+^ fibroblasts. In keeping with this model, no evidence was detected to support extracellular secretion of Minion/Myomerger [7].

A working model that Myomerger–Minion is membrane tethered highlights the need to decipher its membrane topology. Although further experimentation with different epitope-tagged variants is required, a FLAG epitope engineered on the C-terminus of the protein was detectable by immunostaining on live cells, suggesting this region is extracellular [9]. Furthermore, Minion–Myomerger–FLAG is functional as it is able to rescue fusion of Minion–Myomerger knockout myoblasts, and a zebrafish ortholog containing a shorter C-terminal sequence is also functional [7, 9]. Taken together, this indicates that the C-terminal amino acids of Minion–Myomerger do not dramatically impact function. Finally, it is worth noting that Myomerger–Minion also contains potential sites for modifications that include lipidation, glycosylation, and phosphorylation (Millay, Schmedt, and Sampath, unpublished observations). Additional work will be required in order to fully understand how these post-translational modifications are temporally and spatially regulated to Minion–Myomerger to induce fusion.

## Myomaker and Myomerger–Minion function

Perhaps, the most important questions about Myomaker and Minion–Myomerger are their mechanisms of action, specifically whether they participate in the same or different aspects of the fusion process, and how their roles may be integrated into the known pathways that govern fusion. The two main pieces of data that argue for a shared function through forming complexes are the reported interaction between epitope-tagged versions of these proteins when co-overexpressed [9], and the fact that, from a global view, Myomaker knockout and Myomerger–Minion knockout embryos exhibit a similar phenotype (differentiated but unfused myocytes). With regard to the first point, the posited physical interaction has not been observed by IP-mass spectrometry under conditions that successfully recovered other specific interacting proteins [7]. Although negative findings should not be over-interpreted, these data are consistent with the lack of co-localization of the two proteins in intracellular compartments as determined by immunofluorescence [8].

With regard to the second point, subtle differences exist between the phenotypes of Myomaker and Minion–Myomerger knockout mice, which could provide insight into distinct functions of each protein. For instance, Minion–Myomerger KO mice displayed more myocytes, with organized sarcromeres, at E17.5. Moreover, it was observed that myocytes within a particular muscle are more closely aligned in Myomerger–Minion KO embryos. While direct side-by-side comparisons are needed to confirm these, and identify new, phenotypic differences, the available functional data also suggest distinct functions. This includes reconstitution experiments demonstrating an asymmetric requirement for the two proteins in the fusion pair, the finding that Myomerger–Minion is not required for Myomaker function, and the fact that neither protein is required for expression or appropriate localization of the other. Given the current biochemical and functional data, our interpretation is that Myomaker and Myomerger–Minion participate in distinct aspects of the fusion program. Whether a physical interaction exists and if so how it is regulated and functions will no doubt be resolved through continued experimentation in additional labs and by the development of more sensitive and robust antibody reagents.

## Relationship with known myoblast fusion pathways

Current models of myoblast fusion in multiple systems indicate a three-step process in which cells first recognize and adhere to each other, followed by induction of close membrane apposition through F-actin protrusions, and ultimately fusion pore formation through lipid bilayer rearrangement. Some components of this process do not appear highly conserved between vertebrates and invertebrates. For instance, the Ig superfamily of adhesion proteins involved in myoblast fusion in *Drosophila* are well defined (Dumbfounded and Roughest on founder cells and Sticks and stones in fusion competent myoblasts), whereas the adhesion proteins necessary for vertebrate myoblast fusion are not clear [20]. Jamb and Jamc are important in zebrafish but not in the mouse, and the other major class of candidate adhesion molecules in the mouse, cadherins, are similarly dispensable in vivo [20]. Compensation between families of adhesion proteins may partially explain this discrepancy, but nonetheless, there is clear divergence between the effectors of adhesion in *Drosophila* and vertebrates.

In contrast, the role of the actin cytoskeleton appears more well conserved. Elegant work in *Drosophila* embryos showed that invasion of F-actin protrusions are necessary for fusion, potentially to enhance membrane proximity [21]. While these structures were not visualized during fusion of *Drosophila* flight muscles [22], many of the components of the actin remodeling machinery appear functionally conserved to zebrafish and mice. These include homologs of the Arp2/3 activator N-WASP [23], the GTPase Rac1 [24], and Rac-activating GEFs of the Dock family [25, 26]. More specific to vertebrates was the finding that the GPCR BAI3 is also required for myoblast fusion and functions by activating the Rac1 pathway through ELMO–Dock [27].

IP-mass spectrometry studies of Minion–Myomerger in differentiating C2C12 myoblasts recovered many interacting proteins with annotated functions in cytoskeletal function [7]. Among these was the Ferlin family member Dysferlin, which is mutated in a class of inherited muscular dystrophies known as dysferlinopathies. Dysferlin has been shown to have a number of roles in regulation of membrane repair, vesicle trafficking, and exocytosis [28]. All of these processes have been implicated in myoblast fusion, and dysferlin deficiency has indeed been shown to inhibit myoblast fusion [29]. Dysferlin loss notably results in loss of multinucleated myofibers with accumulation of binucleated cells, a feature shared in common with loss of Myomaker, Myomerger–Minion, and the *Drosophila* adhesion regulator rolling pebbles [30, 31] and Dock1 homolog myoblast city [32].

The fusion induced by co-expression of Myomaker and Myomerger/Minion is strongly dependent on remodeling of the actin cytoskeleton and can be blocked by inhibitors of actin polymerization [6, 7]. While it is still unclear whether and how these proteins may activate actin polymerization, one intriguing observation is that heterologous induction of fusion in fibroblasts is associated with accumulation of bands of F-actin paralleling the plasma membrane [7]. These appear similar to structures known variously in other systems as actin walls or actin sheaths. Formation of the actin wall in fusing murine myoblasts requires the function of non-muscle myosin type IIA [33], which also participates in formation of the actin sheath in *Drosophila* founder cells [21]. These structures have been speculated to provide mechanical resistance for the invasion of membrane extensions from the fusion partner, e.g., the podosome-like structure (PLS) of the *Drosophila* fusion competent myoblast [21]. Whether cell fusion induced by expression of Myomaker and Minion–Myomerger likewise requires activity of myosin IIA remains to be determined.

A potentially related pathway that has recently been implicated in mammalian myoblast fusion involves the role of phospholipid membrane constituents such as phosphatidylserine (PS). Under normal conditions, PS is asymmetrically distributed to the cytoplasmic face of the plasma membrane but can be translocated to the extracellular face in a regulated manner, for instance during apoptosis. It has long been known that myoblast differentiation leads to a non-apoptotic transient exposure of PS that could serve as a signal directly promoting the fusion process [34]. Whether apoptotic cells directly regulate myoblast fusion through their abundance of extracellular PS as has been previously proposed [35] requires further investigation. Of note, transient exposure of PS independent of apoptosis is also associated with fusion of trophoblasts, osteoclasts, and during virus-cell fusion [36–38]. How PS is actively translocated to the extracellular surface of myoblasts is not completely understood, although Xkr8 is a candidate for this activity [39]. In myoblasts, PS acts as a ligand for specific cell surface receptors, including BAI1 [35] and Stabilin-2 [40]. Both BAI1 and Stabilin-2 function to activate Rac1 via the ELMO/Dock1 pathway. Given that BAI1 and Stabilin-2 are membrane proteins and activate branched chain actin polymerization, it is tempting to speculate that they act in parallel to Myomaker and Minion–Myomerger. One clear difference though is that whereas knockout of the latter proteins is embryonic lethal with failure of muscle formation, loss of BAI1 and/or Stabilin-2 gives rise to a relatively mild phenotype most evident following injury [35, 40]. This may in part reflect mechanistic differences between myoblast fusion in developmental and regenerative contexts.

Ultimately, the fusion of membranes requires a lowering of the thermodynamic barriers to bring cell membranes in close proximity and rearrange phospholipids. This is classically achieved through proper expression and localization of proteins that remodel the membrane [41]; however, the precise membrane reorganization events that drive myoblast fusion are not clear. At a membrane level, current evidence obtained through a synchronized fusion assay indicates that myoblast fusion proceeds first through the fusion of the outer membranes (hemifusion) followed by pore formation and expansion [42]. Interestingly, hemifusion and pore formation are regulated by distinct proteins. Extracellular annexins (a receptor for PS) are involved in hemifusion, while pore formation requires dynamin activity, cell metabolism, and phosphatidylinositol(4,5)bisphosphate [42]. Based on their transmembrane nature and sufficiency to fuse normally non-fusing cells, it is enticing to speculate that Myomaker and/or Myomerger/Minion are central mediators of the necessary membrane remodeling events that drive fusion.

## Comparison to viral fusion systems: FAST

Given the complexity of mammalian myoblast fusion and the multitude of mechanisms involved, the minimal “two-component” system for fusion induction using Myomaker and Myomerger–Minion has value as a tool for understanding which pathways are core and which are secondary or regulatory. Viruses have likewise evolved simplified systems to drive cell fusion, and comparison to these proteins may help to illuminate the key features of mammalian myogenic fusion regulators.

Viral fusogens can be separated into structural and non-structural proteins. The structural fusion proteins are large surface components of enveloped viruses, serve to enhance viral entry, and are not considered further here. In contrast, the non-enveloped viruses of the reovirus family encode a group of small membrane-associated proteins which primarily function to drive target cell syncytialization through cell-cell fusion. These fusion-associated small transmembrane (FAST) proteins do not function in viral entry but are thought to aid in lateral spread within tissue, with the degree of syncytialization correlating with virulence [43, 44]. Like Myomaker and Minion/Myomerger, FAST proteins are capable of fusion induction when exogenously expressed in cells such as fibroblasts [45]. An obvious difference between FAST fusion and the Myomaker–Myomerger–Minion fusion system is that FAST proteins are modular fusogens, containing all the required membrane-altering events that drive fusion. In contrast, neither Myomaker nor Minion–Myomerger alone are sufficient for fusion. This bipartite myoblast fusion system suggests that the functions required for fusion have been delegated to two factors, perhaps to allow more regulatory control of the fusion process.

A typical FAST protein (e.g., Baboon orthoreovirus p15) contains an N-terminal myristoylation site, a transmembrane (TM) domain, and an endodomain containing a polybasic motif (PM) and hydrophobic patch (HP). The polybasic motif functions as a Golgi export signal for membrane targeting [46], whereas the hydrophobic patch is thought to pack against and stabilize highly curved nascent fusion pores in order to facilitate pore expansion [47]. Comparison to the domain structure of the mammalian myogenic fusion regulators is revealing. Myomaker contains a consensus N-terminal myristoylation site (not absolutely required for function) and is also palmitoylated, Myomerger–Minion contain consensus palmitoylation sites, Myomaker contains multiple transmembrane domains, and Minion/Myomerger contains an N-terminal hydrophobic domain as well as potential amphipathic helices. These structural and functional features suggest that FAST proteins and the Myomaker–Minion–Myomerger system may have convergently evolved for a similar function in cell fusion.

This model is useful in that it makes testable predictions that may help elucidate the mechanism of Myomaker and Myomerger–Minion activity. For instance, the hydrophobic and amphipathic amino terminus of Minion/Myomerger may be analogous to the hydrophobic patch of FAST proteins and may function to lower the energy barrier for formation and expansion of fusion pores. Indeed, this possibility is supported by the observation that the p15 HP can be replaced by a heterologous FAST amphipathic helix [47]. Likewise, lipidation of Myomaker and Myomerger/Minion may be essential for membrane targeting and fusogenic function, as has been shown for multiple FAST proteins [48–50]. The precise evolutionary relationship (if any) between these functionally similar proteins also deserves further examination.

## Summary

**Fig. 1:**
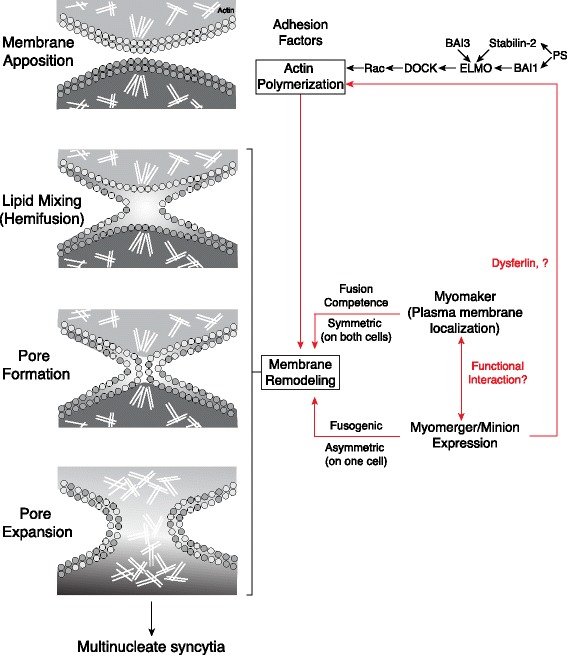
Schematic representation of the stages of vertebrate myoblast fusion. Membrane apposition requires cell adhesion and remodeling of the actin cytoskeleton, which is controlled in part by signaling via phosphatidylserine (PS) receptors and BAI3. Myomaker and Minion/Myomerger are proposed to act at later stages, through control of membrane remodeling events that together define fusion competence, drive fusion pore formation, and support pore expansion. Myomaker and Minion/Myomerger also define potential asymmetry in the fusion process and could require an interaction between the two proteins for fusion. The black arrows indicate pathways supported by experimental evidence, whereas red arrows depict proposed functions

Taken together, the findings discussed above support a model in which Myomaker expression defines the fusion competence of differentiating muscle precursors, whereas Minion–Myomerger functions to activate fusogenicity potentially through fusion pore formation and/or expansion (Fig. 1). The participation of other more ubiquitously expressed factors, such as components of the actin remodeling machinery, is also required, although the precise mechanism leading to activation of these pathways remains incompletely understood. The many similarities to the fusogenic FAST proteins on non-enveloped viruses suggest convergent evolution to solve the problem of temporally and spatially regulated membrane fusion.

## Therapeutic potential of targeted cell fusion

The identification of a minimal system for induction of cell fusion opens the door for potential translational applications. For instance, cell fusion has long been suggested as a potential approach for the complementation of genetic deficiencies in vivo. This model has been most clearly articulated in the context of Duchenne muscular dystrophy (DMD), in which mutation in the *Dystrophin* gene leads to progressive myofiber injury, impaired muscle regeneration, and eventual death. Early work in the *mdx* mouse model of DMD suggested that exogenously delivered cells arising from either the muscle or hematopoietic lineage could contribute to myofiber formation via cell fusion [51, 52]. This could in turn lead to localized restoration of Dystrophin expression. Intriguingly, some variations on this approach included systemic rather than intramuscular cell administration, suggesting that whole-body complementation could in theory be achievable. Importantly though, the degree of myofiber contribution achievable by this method was extremely low, suggesting that restoration of muscle function would be unlikely without significant improvements in delivery.

The identification of Myomaker opened the door to increasing the fusogenic potential of cell-based therapies, thereby increasing their complementation potential. Studies utilizing non-muscle-derived cells, such as fibroblasts and mesenchymal stem cells, indeed demonstrated that overexpression of Myomaker could promote the fusion of these cells into the muscle in vivo [53]. It remains unclear whether this method could be used to boost myoblast fusion to clinically relevant levels. Nevertheless, it seems likely that the combination of Myomaker and Minion–Myomerger expression should further significantly boost fusogenic potential, if it is possible to prevent the premature fusion of these cells with one another.

In addition to skeletal muscle development and repair, cell fusion has been reported to occur in a number of physiological and pathophysiological settings. Inflammation appears to be a critical factor in the development of a permissive environment for fusion, and it has been observed that exogenous bone marrow-derived cells extensively fuse to cerebellar Purkinje neurons in experimental models of multiple sclerosis [54]. Moreover, bone marrow-derived cells, and in particular macrophages, have been shown to suppress the lethality of mice lacking the fumaryl acetoacetate hydrolase gene *Fah*, a preclinical model of hereditary tyrosinemia type I; this occurs via fusion of donor cells with Fah-deficient hepatocytes, thereby restoring hepatocyte viability and function [55]. If, as speculated, Minion/Myomerger is involved predominantly in the downstream processes of fusion pore formation/expansion, its overexpression may dramatically improve the efficiency of cell fusion and therefore functional complementation in such settings.

Additional opportunities exist for the therapeutic application of targeted fusion to cancer. Cancer vaccines based on tumor-dendritic cell heterokaryons can potently induce immunity through the enhanced presentation of tumor antigens via both class I and class II MHC [56], and co-expression of Minion–Myomerger and Myomaker could dramatically improve the efficiency of forming these hybrids. Similarly, lack of viral spreading represents a fundamental limit to tumor lysis in oncolytic virus therapy [57], and co-delivery of fusogens correspondingly increases their efficacy [58–60].

Finally, the heterologous fusion systems discussed here promise to be equally useful in basic research related to cell fate and reprogramming. Early studies on somatic cell nuclear reprogramming relied on chemical methods such as PEG treatment to induce heterokaryon formation [61], an inefficient and poorly controlled process. In contrast, transient expression of Myomaker and Minion–Myomerger via conditional/inducible systems will allow far greater control of fusion, with resulting insights into the temporal dynamics of nuclear reprogramming.

## Conclusions

The discovery of the first two mammalian muscle-specific fusion factors, Myomaker and Minion–Myomerger, marks the beginning of a new chapter in the study of myoblast fusion. Tools now exist to dominantly induce fusion, allowing detailed mechanistic analysis as well as identification of additional novel players. While these studies are only beginning, the existing data have already shed new light on unexpected similarities between fusion in various systems and may lead to further unification of our understanding of fusion in vertebrates and invertebrates. Given the numerous opportunities for clinical application of cell fusion, as well as a recent report demonstrating human mutations in this pathway as a cause of congenital myopathy, we anticipate that our expanding knowledge of the fundamental mechanisms of cell fusion will translate into novel opportunities to positively impact human health.

## Abbreviations

Eff-1: Epithelial fusion failure
FAST: Fusion-associated small transmembrane
HP: Hydrophobic patch
MRFs: Myogenic regulatory factors
MuSC: Muscle stem cells
PLS: Podosome-like structure
PM: Polybasic motif
PS: Phosphatidylserine
smORF: Small ORF
TM: Transmembrane
